# Towards a circular economy: valorization of banana peels by developing bio-composites thermal insulators

**DOI:** 10.1038/s41598-023-37994-1

**Published:** 2023-08-07

**Authors:** Gehad R. Mohamed, Rehab K. Mahmoud, Mohamed Shaban, Irene S. Fahim, H. M. Abd El‑Salam, Hamada M. Mahmoud

**Affiliations:** 1 Faculty of Architecture and Design Innovation, Victoria University of Wellington, Wellington, New Zealand; 2 Chemistry Department, Faculty of Science, Beni-Suef University, Beni-Suef City, Egypt; 3 Nanophotonics and Applications Lab, Physics Department, Faculty of Science, Beni-Suef University, Beni-Suef, Egypt; 4 Departmentof Industrial Engineering, School of Engineering, SESC Reseaarch Center, The Nile University, Nile Avenue. Giza, 116453 Egypt; 5 Department of Chemistry, Faculty of Science, Polymer Research Laboratory, Beni-Suef University, Beni Suef, 62514 Egypt; 6 Zoology Department, Faculty of Science, Beni-Suef University, Beni Suef, Egypt

**Keywords:** Environmental sciences, Energy science and technology

## Abstract

The building construction materials are responsible for a large amount of energy and natural resource consumption. In light of the current challenges of resource scarcity and global climate change, the circular economy (CE) is a promising strategy to mitigate pressure on the environment, improve supplying of raw materials, and increase new market and employment opportunities. Developing eco-friendly thermal insulation materials based on agro-waste is a new waste management trend to achieve the sustainability of the resource and energy consumption in the construction sectors. In this work, banana-polystyrene composites were prepared by mixing the banana peels powder (BP) with polystyrene (PS) in different weight ratios (90:10, 80:20, 70:30, and 60:40). The physical and thermal properties such as thermal conductivity, electrical conductivity, Fourier Transform Infrared (FTIR), crystallographic structures of the fibers, X-Ray Diffraction (XRD), Thermogravimetric Analysis (TGA), and Differential Scanning Calorimetry (DSC) were carried out on BP and BP-PS1 that were prepared with ten wt.% and 20 wt.% of polystyrene powder (BP-PS2). The bio-composites results showed low thermal conductivity ranging from 0.028 to 0.030 W/m.K. The BP-PS2 exhibited a lower thermal conductivity of 0.027 W/m.K, while the pure peel powder demonstrated notable thermal stability, indicated by a total weight loss of 66.4% and a high crystallinity value of 56.1%. Furthermore, the thermal analysis (TGA) and X-Ray Diffraction (XRD) demonstrated that the pure banana peel has the highest thermal stability and crystallinity. These findings indicate that using banana peel-polystyrene composites represents an innovative solution for thermal insulation in buildings as an alternative to conventional materials to reduce energy and resource consumption.

## Introduction

The construction industry is responsible for several negative effects^1^. In order to reduce the ecological effects, economic losses, and resource consumption, thermal insulators play a crucial role in lowering the heating and cooling load by reducing the heat transmission through the building envelope^2^. Several insulating materials are commercially available such as fiberglass, urethane, cork, and Extruded Polystyrene^3^. However, the environmental concerns related to the manufacturing process of conventional thermal insulators justify the need to develop materials with eco-friendly features^4^.

Conversely, agricultural waste disposal is also a significant problem in developing countries. For instance, in Egypt, over 39 thousand metric tons of vegetal waste was reported (Vegetal waste, production (thousand metric tons) for Egypt–Tilasto.) the most common strategies to manage agriculture waste are composting, dumping in landfills, and open-air incineration, which as a result cause severe environmental concern^5^. Nonetheless, new trends have shown that using agricultural waste in construction materials manufacturing is a feasible solution to tackle the identified challenges^6^.

Utilizing thermal insulation materials from agricultural waste could reflect the circular economy concept as an optimum solution to reduce waste generation and energy consumption^7^. Exploiting natural fibers as green raw materials produces thermal insulators that are non-toxic, recyclable, locally available, and characterized by a low carbon footprint and lower energy consumption of the manufacturing process^2^. On the other.hand, using natural by-products as thermal insulators has drawbacks such as nonresistant fire, the Influence of weather, and Lower durability^8^. In order to assess the suitability of thermal insulator production, several researchers studied the thermal performance and chemical characteristics of different natural fibers. The thermal conductivity values were found to range between 0.034 W/m.K and 0.042 W/m.K for sugarcane bagasse waste fibers^9^, 0.050 and 0.132 W/m.K for oil palm wood^10^, 0.0393–0.0478 W/m.K for rice straw^11^.

The banana crop is one of the most significant crops in the world. In 2019, the banana production was more than 116 million tons globally, and around 1.3 million tons in Egypt^12^. Banana peels are an abundant waste for every 10 tons of bananas, one ton of waste is produced^13^. Banana peel is discarded as a useless material, and it represents about 40% of the Banana total weight, and this causes waste management problems because of its phosphorus and nitrogen content^14^. Banana peel contains 20–30% fiber and is rich in carbohydrates, proteins, dietary fibers, and mono and disaccharides, making this waste a promising source for the industry^15^. The banana peel is a low-cost, readily available, lightweight, eco-friendly material and could be transformed enzymatically, physically, and chemically into value-added products^16^. To produce green building materials, several researchers investigated the thermal conductivity of banana composites. The composite's thermal conductivity ranged from 0.245 to 0.363 W/m.K for banana and jute fiber^17^, 0.149 W/m.K for banana-solid waste^18^, 0.0183 to 0.03168 W/m.K for Banana leaves and polystyrene^19^, 0.6 W/m.K for banana, bagasse and cement brick^20^ and 0.3880 to 0.04495 W/m.K for banana fiber^21^.

Polymers are categorized into thermosets and thermoplastics^22^. Polystyrene (PS) is a synthetic thermoplastic polymer fabricated from styrene. Recently, polystyrene was used as an essential material in thermal insulation panels composition due to its low cost, low water absorption, low density, low acoustical absorption, and low thermal conductivity 0.038 W/m K Though the expanded polystyrene (EPS) products have 35% of the total insulation market in Europe, it has numerous drawbacks, including toxicity, flammability, and poor durability^23^.

The circular economy principles could be achieved by closing the loop of agriculture materials consumption. This involves valorizing agricultural wastes, giving them another life, and utilizing them across different industries, rather than disposing of them in the landfill. The comprehensive literature review revealed that information is scarce regarding Egyptian agriculture waste, including banana peel characterizations and potential applications in the construction industry. Geographically, it cannot not rely on the thermal properties of banana waste in other regions of the world due to the impact of climatic conditions, soil nature, and water quality on the biological features of the banana tree. This includes the cellulose and lignin content which directly affect the physical features and the thermal conductivity properties. Consequently, this will affect its efficiency for insulating properties^24^.

**Figure 1:**
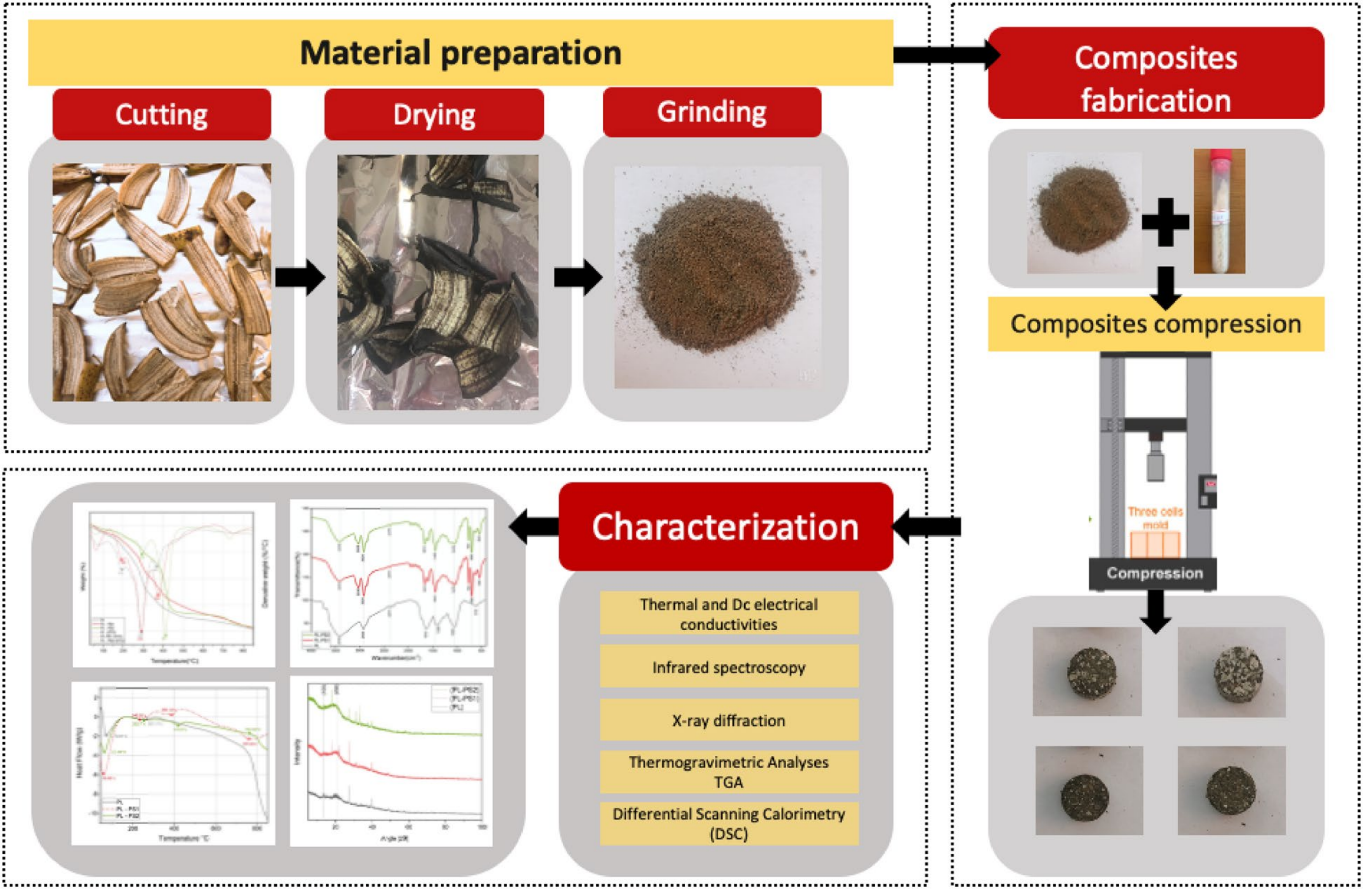
Fabrication of bio-composites thermal insulators using banana peels.

The present study aims to manufacture thermal insulating material based on a banana peel fiber. Different composites will be prepared by adding a polystyrene filler to banana peel powder, to optimize the thermal characterization of the prepared composites. Therefore, the pure banana peel and the prepared composites will be subjected to thermal conductivity, Infrared analysis (FT-IR), X-ray Diffraction (XRD), thermogravimetric analysis (TGA), and differential scanning calorimetry (DSC) to assess the possibility of the tested banana peel composite to produce an economical insulation material. A summary of the preparation process and the findings are presented in the graphical abstract (Fig. 1).

## Materials and methods

### Sample collection and preparation

**Figure 2:**
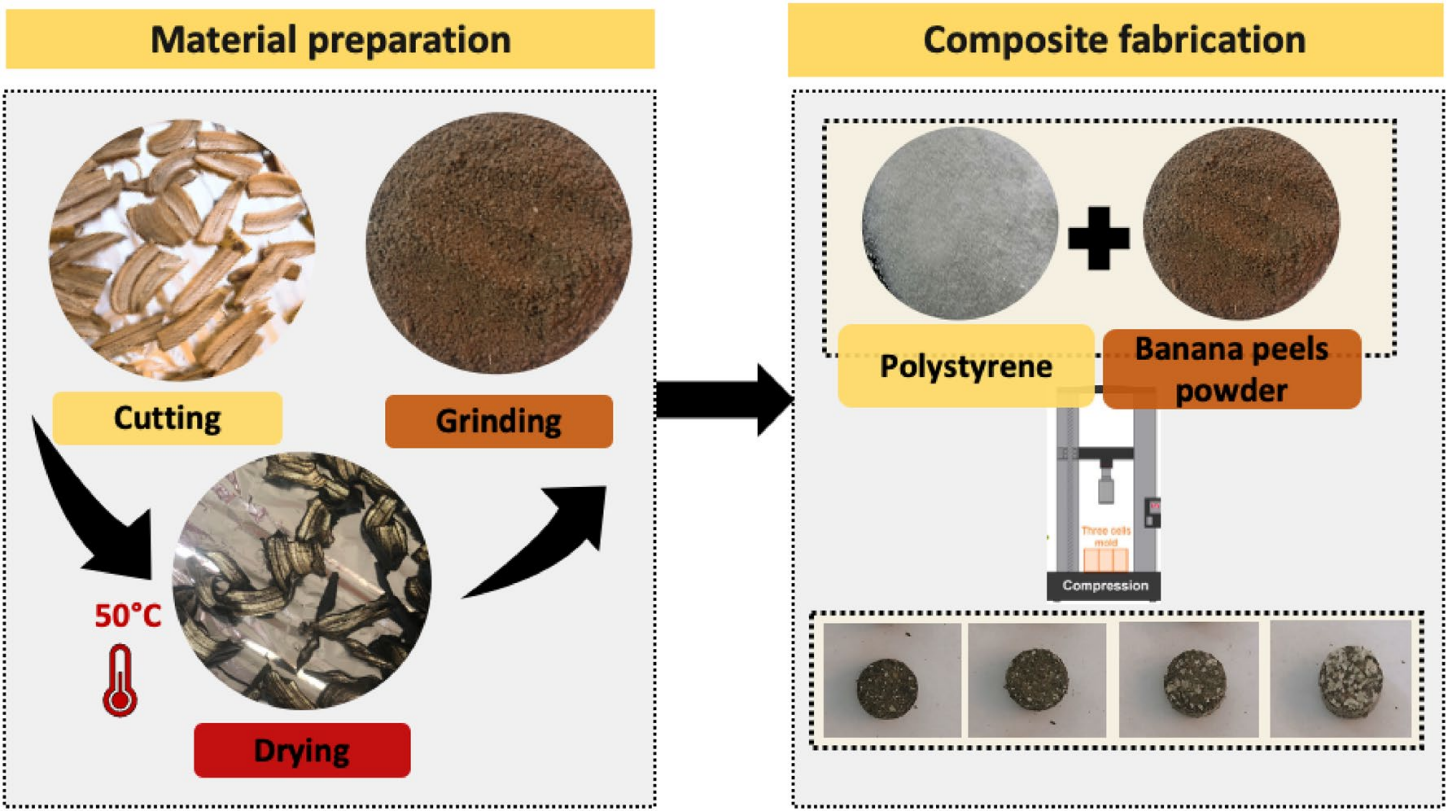
The production process of the Banana peels-polystyrene composites.

The banana fruit (peels) used were obtained from an available local fruit market, Beni-suef, Egypt. As seen in Fig. 2, the banana peels were cut into small pieces (4–5 cm), and then the samples were dried in the open air for five days; after that dried in the laboratory oven at 50 °C for 4 h (T 6060 Heraeus instruments) to remove moisture content. Finally, the dried peels were ground by a home blinder and manually sieved to get fine powder, then sealed and stored in the laboratory at room temperature ^25^.

### The polymer preparation

Polystyrene (PS), which is one of the largest volume vinyl polymers, was synthesized by free radical oxidation polymerization of a volume of 5 ml styrene free from inhibitor that was dissolved in a book of 20 ml dimethylformamide (DMF), using 5 gm of ammonium persulfate dissolved in 10 ml distilled water at 60 °C under nitrogen for 3 h. PS was filtered at room temperature after 12 h., washed with water/DMF mixture, and dried under vacuum at 60 °C.

### Composite fabrication

To produce the banana-polystyrene composites (BP-PS) with different filler contents, the polystyrene was added to banana peel powder with varying ratios of 10%, 20%, 30%, and 40% weight PS (Table 1). Next, the composites were compressed using a hydraulic press at 200 Pascal to produce cylinder pellets weighing 0.5 g, diameter of 1 cm and thickness of 0.5 cm. Then, the composite's Thermo-Physical properties were assessed, and the results were averaged and reported^23^.

**Table 1:** Samples abbreviation.

Samples	Banana Peels powder (wt. %)	Polystyrene (wt. %)
BP	100	0
BP-PS1	90	10
BP-PS2	80	20
BP-PS3	70	30
BP-PS4	60	40
PS	0	100

### Characterization

#### Thermal conductivity

**Figure 3:**
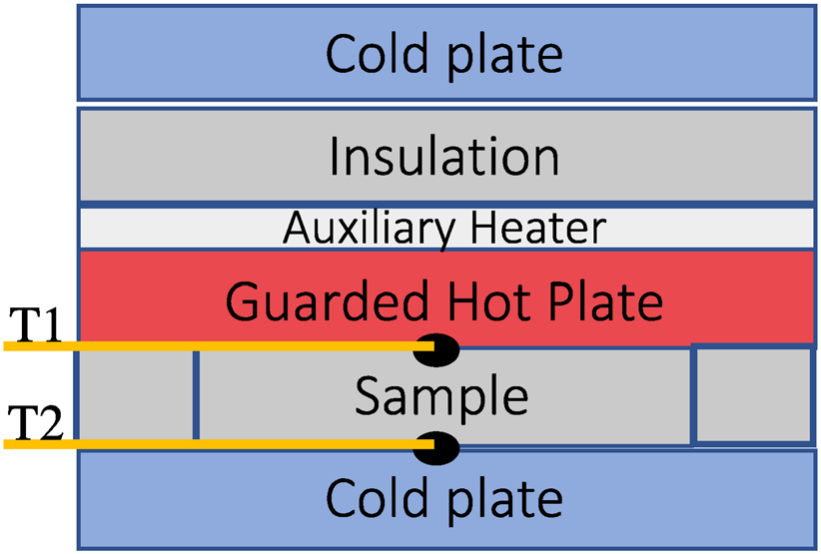
Schematic diagram of the used experimental set-up for measuring the thermal conductivity of the composites.

From Fig. 3 thermal conductivity of all samples was measured using a homemade apparatus that allows the thermal conductivity measurement at temperatures up to 25 C^26^. As shown in Fig. 3. the apparatus comprises two Peltier cold plates, thermal insulators, an auxiliary heater, temperature sensors, and an electrically guarded hot plate. This configuration permits heat flow through the samples in an adiabatic manner^27^. Based on the steady-state technique, thermal conductivity was measured by applying the Fourier-Biot law of heat conduction^28^. The thermal conductivity coefficients were measured for pure banana peels, polystyrene, and all prepared BP-PS composites. The Keithley measurement source unit measured the DC current–voltage characteristics of the samples (Model 2400). The DC conductance of the samples was estimated from the measured DC current–voltage characteristics.

#### X-Ray Diffraction (XRD)

BP, BP-PS1, and BP-PS2 crystallographic nature were examined by the XPERT-PRO X-Ray Diffractometer, equipped with Cu radiation (λ = 1.540 Å) source operated at a tension of 40 kV and current of 40 mA. XRD data were recorded within the range of scattering angles (2θ) of 10 to 90° at room temperature. The BP, BP-PS1, and BP-PS3 Crystalline index (CI) and crystallinity (Cr) were evaluated by Segal methods using Eqs. (1) and (2),

1 $$CI\;(\% ) = \frac{{\left( {I_{c} - I _{am} } \right)}}{{I_{c} }}*100$$

2 $$Cr\;(\% ) = \frac{{I_{c} }}{{I_{c} + I_{am} }}*100$$

It corresponds to the maximum peak intensities of crystalline regions, and Iam corresponds to the amorphous areas^29^.

#### Fourier-transform infrared spectroscopy analysis

For FT-IR analysis, the specimens were recorded by VERTEX 70 FTIR spectrometer within a wavenumber range of 4000 to 500 cm^−1^ at room temperature.

#### Thermal stability

The SDT Q600 V20.9 Build 20. An instrument was used to perform the thermal analysis. Under nitrogen flowing at 20 ml/min, A heating rate of 10 °C/min was used to increase the temperature from 30 to 850 °C^23^.

## Results and discussion

### Thermal and DC electrical conductivities

**Figure 4:**
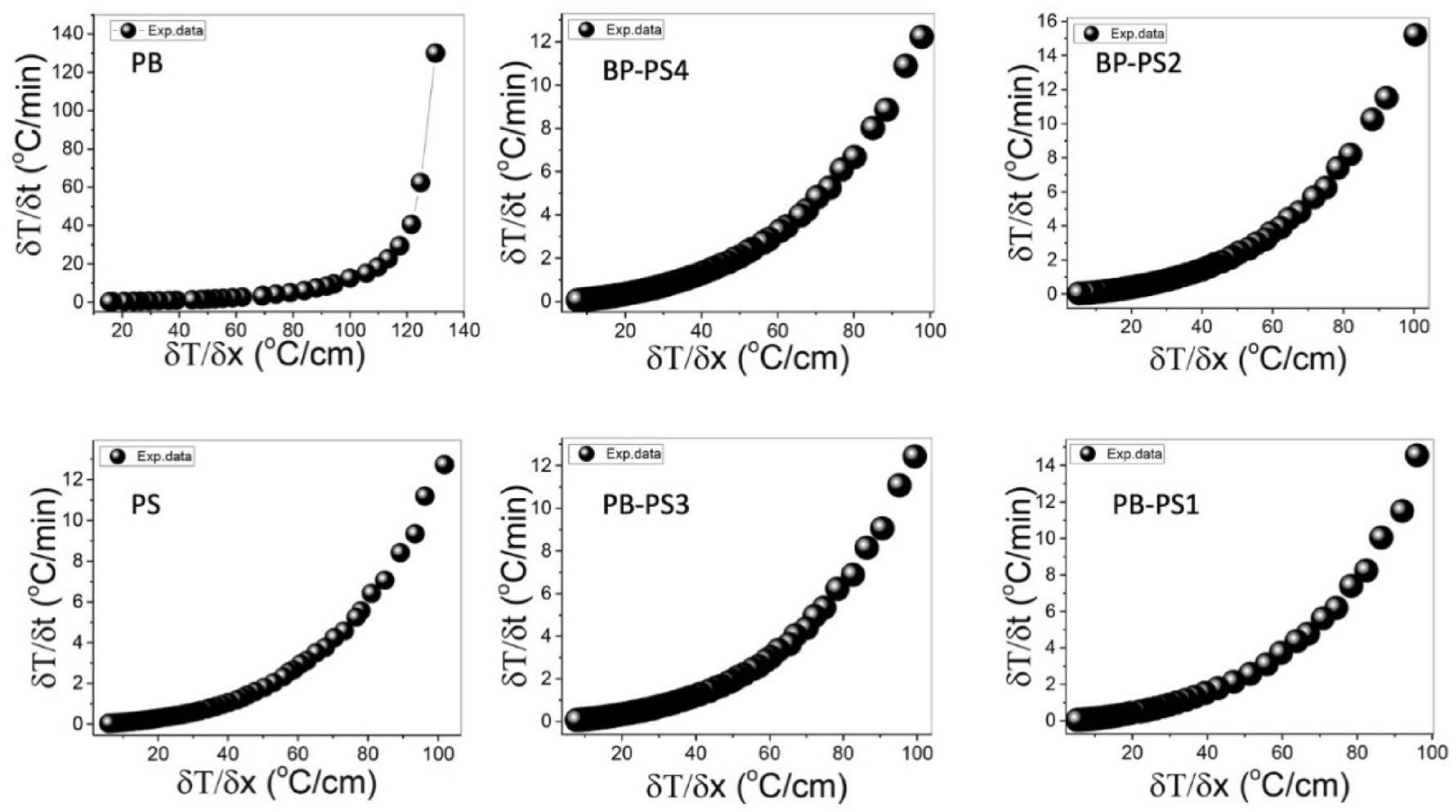
The rate of change of the temperature (δT/δt) versus the temperature gradient ((δT/δx) of the different samples (PS: polystyrene, BP: Banana Peel).

The Fig. 4 shows the rate of temperature change (δT/δt) versus the temperature gradient (δT/δx) of the different specimens measured using our homemade apparatus (Fig. 3). The thermal conductivities of the prepared composites are shown in Fig. 4. were calculated as average values and are presented along with the standard deviation in Table 2. The thermal conductivities of banana peel (BP) and pure polystyrene (PS) were 0.132 and 0.022 W/m.K, respectively. The composites (10–40% polystyrene) had thermal conductivity coefficients ranging from 0.028–0.030 W/m.K. After adding PS, there was a trivial variation in the thermal conductivity coefficients relative to that of pure banana peel among the four composites BP-PS1, BP-PS2, BP-PS3, and BP-PS4 (i.e., decrease of 78,7%, 79.5%, 77.2%, and 78.0%, respectively). The difference in the thermal conductivities of the banana fibers and polystyrene explained the decrease in the thermal conductivity of the composite. The results support the role of adding polystyrene to improve the thermal conductivity performance of banana composites, according to Al-Kadhemy (2013)^30^, who stated that the material is qualified to be a thermal insulator when its thermal conductivity is less than 0.1 W/m.K. The thermal conductivity coefficient of pure banana peels was lower than corn stalk and gypsum (0.1999–0.1 W/m.K) (Binici, Aksogan, and Demirhan 2016) date and polystyrene (0.053 W/m.K)^31^ and camilina and expanded perlite (0.082 W/m.K)^32^, and rice straw and gypsum (0.2–011 W/m.K)^33^ . Meanwhile, the thermal conductivity coefficient of banana peel was higher than banana leaves and polystyrene (0.0183 W/m.K)^19^. Moreover, the BP-PS thermal conductivity coefficients were within the same range as those of conventional insulating materials such as rock wool (0.044 W/m.K), and sheep wool (0.038–0.054 W/m.K)^34^.

**Table 2:** The values of the thermal and electrical conductivities of the BP, PS, and BP-PS composites of different ratios.

Samples	Composite ratio	Thermal conductivity (W/m.K )	Electrical conductance (A/V)
BP	Banana peels are 100%	k = 0.132 ± 0.03492	5.8621E − 8 ± 1.5701E − 10
BP-PS1	BP 90%–PS 10%	k = 0.028 ± 0.00752	1.628E − 9 ± 1.930E − 11
BP-PS2	BP 80%–PS 20%	k = 0.027 ± 0.00815	5.821E − 9 ± 1.991E − 11
BP-PS3	BP 70%–PS 30%	k = 0.030 ± 0.00815	7.186E − 9 ± 2.435E − 11
BP-PS4	BP 60%–PS 40%	k = 0.029 ± 0.00843	7.556E − 10 ± 1.629E − 11
PS	Pure Polystyrene 100%	k = 0.022 ± 0.00526	7.761E − 10 ± 1.915E − 11

The DC current–voltage characteristics of the banana peel and the prepared composites (BP-PS1, BP-PS2, BP-PS3, and BP-PS4) were evaluated using a Keithly measurement source unit (model:2400). As noticed in the present study, the pure banana peels showed non-ohmic behavior that was tuned to linear Ohmic after the PS was blended. Therefore, the experimental data were linearly fitted, and the electrical conductance of the composites were calculated and are listed in Table 2. All composites (BP-PS2, BP-PS3, and BP-PS4) showed low conductance values, whereas the BP-PS1 composite showed an electrical conductance value (1.628E-9 ± 1.930E-11 A/V). The observed low thermal and electrical conductivities of the optimized composites support their suitability for industrial insulation applications.

### XRD analysis

**Figure 5:**
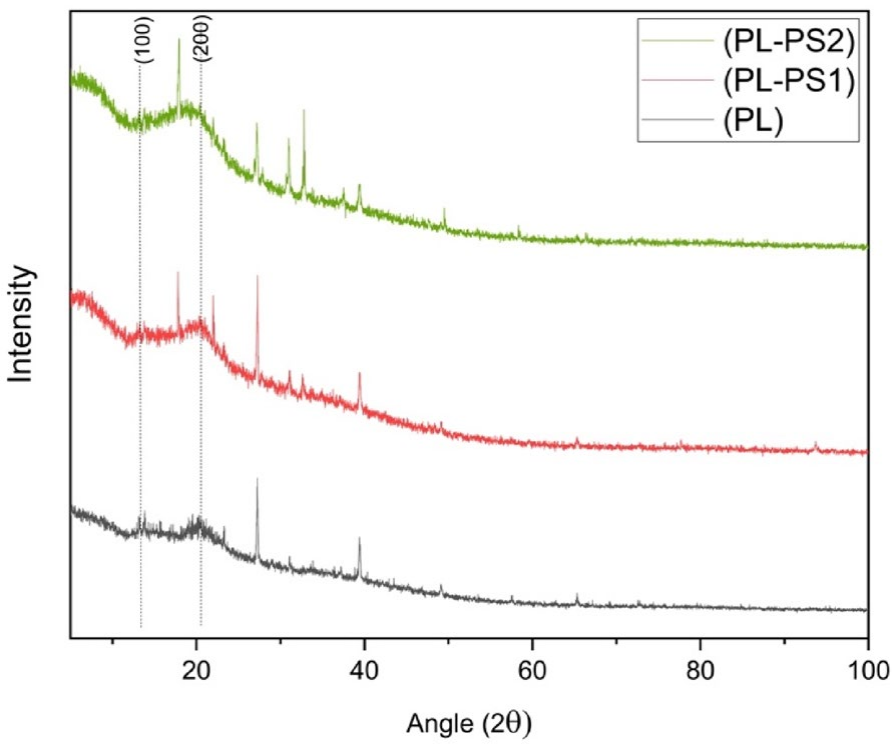
XRD patterns of BP, BP-PS1 and BP-PS2.

In order to better understand the crystallization of banana peel composites, a high crystallinity index is expected to result in stiff, durable, and highly thermal stable fibers^35^**.** The diffractograms obtained for BP, BP-PS1, and BP-PS2 are shown in Fig. 5. The XRD patterns showed similar diffraction peaks. The observed peaks at 2θ = 13.84° (broad) corresponded to (110) crystallographic plane, which indicated the presence of non-cellulosic materials like hemicelluloses and lignin in the fabrics and 21.97° (broad) corresponds to (0 0 2) crystallographic plane which indicated the presence of semi-crystalline cellulose I in the fibers (interplanar distances ''d" of 6.4 Å for 2θ 13.8 and 3.8 Å for 2θ 21.9) (Paulo Henrique Fernandes^36^. The narrow peaks observed in the graphs could be due to contamination present in the fibers, probably from an inorganic material^37^.

The crystallinity of BP was 56.1%, which is higher than the 54% for BP-PS1 and 52.3% for BP-PS2 (Eq. (2)), and the crystallinity index was calculated using Eq. (1) to be 21.8% for BP, which was higher than the 14.9% for BP-PS1 and 9.1% for BP-PS2 (Table 3). The calculated CI values of the composites were lower than that reported for the banana pseudostem by 39%^37^, banana peel-HDPE composite by 65.8% (Paulo Henrique Fernandes^36^.

**Table 3:** The crystallinity index (CI) and crystallinity (Cr) of the BP, BP-PS1, and BP-PS2.

Samples	CI (%)	Cr (%)
BP	21.8	56.1
BP-PS1	14.9	54
BP-PS2	9.1	52.3

### Fourier-transform infrared spectroscopy analysis

**Figure 6:**
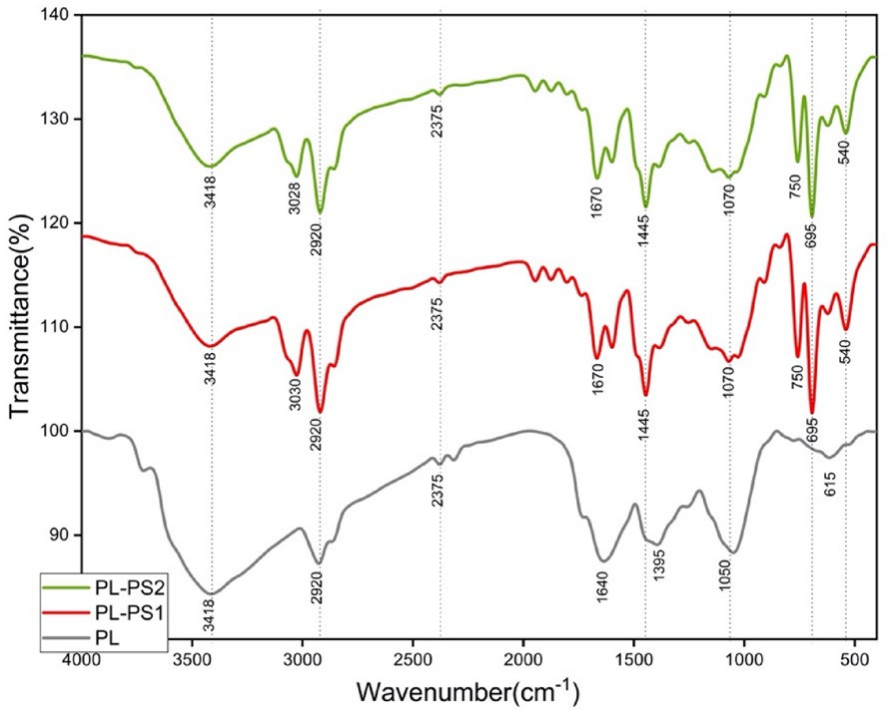
FTIR spectrum of BP, BP-PS1 and BP-PS2.

FT-IR analysis is a beneficial tool in evaluating the composites' chemical composition. It examines the chemical interaction of fiber and polymers, that could affect their physical properties. Moreover, it facilitates the identification of chemical modifications that happened during engineering processes^38^. FT-IR spectra of BP, BP-PS1, and BP-PS2 were obtained to identify the functional groups present in the composites, shown in Fig. 6. The hydrophilicity of the banana peels was reflected in the broad absorption band in the region range from 3700 to 3100 cm^−1^, which was related to the –OH group and intermolecular hydrogen present in their main components (R.^39^. Around 3424 cm^−1^, broadband was observed owing to the stretching vibrations of hydroxyl groups O–H^40^. The peaks observed at 2920 cm^−1^ were due to aliphatic saturated C–H stretching in the hemicellulose and cellulose^41^. The peaks at 1670 cm^−1^ belong to the carbonyl C = O stretching vibration, which corresponded to the carboxylic acid in lignin or the linkage of the ester group in hemicelluloses^29^. The peaks at around 1445 cm^−1^ corresponded to the C = C stretching vibration due to the aromatic ring of lignin^42^. The band in the region of 1070 corresponded to the C–O stretching vibrations of aliphatic alcohol in hemicellulose, cellulose, and lignin found in the peels^39^. The band at 615 cm^−1^ corresponded to the out-of-plane bending vibration of the intermolecular H-bonded O–H group and out-of-plan O–H bending^43^.

After adding polystyrene, different peaks appeared in the region from 1000 to 400, called the aromatic region, owing to the presence of a benzene ring^44^. In addition, the C–H deformation vibration band of benzene ring hydrogen was observed at 750 cm^−1^ due to the polystyrene chemical composition^30^. Table 4 consolidates the central peak positions and assignments of chemical stretching.

**Table 4:** IR regions and vibrations of FTIR spectra.

IR region (cm^−1^)	Vibrations (cm^−1^)	Assignments	Component	Reference
3900–3200	3424	Bonded and non-bonded –OH groups	Cellulose	[50]
3000–2700	2920	C–H stretching (aliphatic–aromatic)	Cellulose and hemicellulose	^41^
1800–1500	1670	C = O Conjugated aromatic rings and carbonyl stretching	Lignin	^41^
1500–1250	1445	C = C Aromatic ring vibrations	Lignin and waxes	^42^
1250–1000	1070	C–O stretching	Hemicellulose, cellulose and lignin	^39^
750–500	615	OH out-of-plane bending	–	^39^
750–500	750	C–H deformation vibration	Polystyrene	^30^

### Thermogravimetric analyses

**Figure 7:**
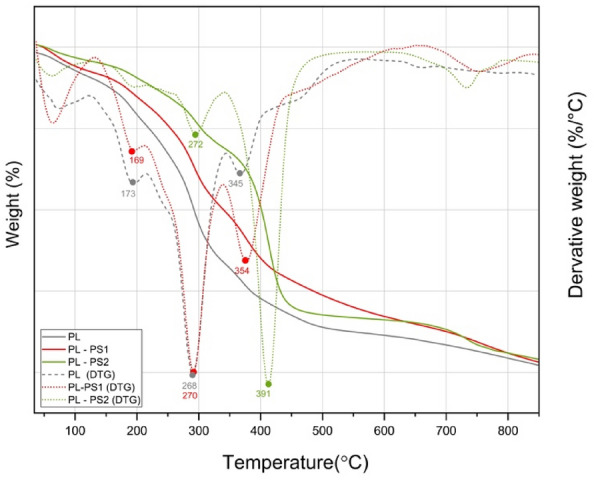
Thermogravimetric analysis (TGA) and derivative thermogravimetric (DTG) curves of the BP, BP-PS1 and BP-PS2.

The thermal stability of the composites was assessed using thermogravimetric analysis (TGA). As depicted in Fig. 7, the TGA and DTG curves show comparable patterns with the four weight loss phases. The first phase of the composite weight loss (30 °C–190 °C) was the evaporation of moisture, which did not show a significant weight loss^39^. The weight loss was 9.68%%, 10.49%, and 6.58% for BP, BP-PS1, and BP-PS2, respectively. This weight degradation could be explained by the loss of moisture content and volatile extractives in the fibers. Although banana peels were dried before the test, the total elimination of the moisture was challenging due to its hydrophilicity nature^39^.

During the second degradation stage, which ranged from 180 to 350 °C, the BP, BP-PS1, and BP-PS2 showed a weight loss of 26.48%, 26.33%, and 16.92% at 268 °C, 270 °C and 272 °C, respectively. The fast degradation of the peel samples could be a result of hemicellulose degradation^29^.

In the third stage (200–400 °C), the samples exhibited significant weight loss (37–50%). This can be attributed to the degradation of α-cellulose, cellulose I, and lignin. The weight loss of BP, BP-PS1and BP-PS2 was 47.26%, 47.57%, and 38.65% at 345 °C, 354, and 391 °C, respectively^45^.

The last stage was lignin decomposition, which was the most challenging component to decompose compared to other organic members. The degradation temperature started from 250 to 800 °C because of its complex structure of aromatic rings composed of heavy cross-linked molecules besides the polysaccharides^46^. The total weight loss of the samples at the temperature of 750 °C was 66.4%, 80.75%, and 81.52% for BP, BP-PS1, and BP-PS2^29^.

The TGA and DTGA profiles of the banana composites showed thermal stability up to 190 °C, which confirms the possibility of using natural composites for insulating applications^47^. The residual weights of the samples obtained at 750 °C are listed in Table.5 shows that BP had the highest residual weight ratio, indicating a higher thermal resistance^48^. The XRD results confirmed that the sample with the highest thermal stability had the highest crystallinity. The addition of polymers lowered the composite stability because of the weak intermolecular bonding of the composites^49^.

**Table 5:** The thermogravimetric analysis results of the BP, BP-PS1, and BP-PS2.

Sample	Peaks	T peak °C	Weight loss %	Residual weight % at 750 °C
BP	1	173	9.68	33.6
	2	268	26.48	
	3	345	47.26	
BP-PS1	1	169	10.49	19.25
	2	270	26.33	
	3	354	47.57	
BP-PS2	1	168	6.58	18.48

. Generally, the increase of the banana peel content in the composites increases the thermal resistance, which, in the case of fire, affects the shrinkage performance of the insulating materials^23^. These results are in agreement with those of Banana leaves and polystyrene^19^, high-density polyethylene banana peel fibers (Paulo Henrique Fernandes^36^, *Musa paradisiaca*^39^, date pit polystyrene composites^23^.

### Differential scanning calorimetry (DSC)

**Figure 8:**
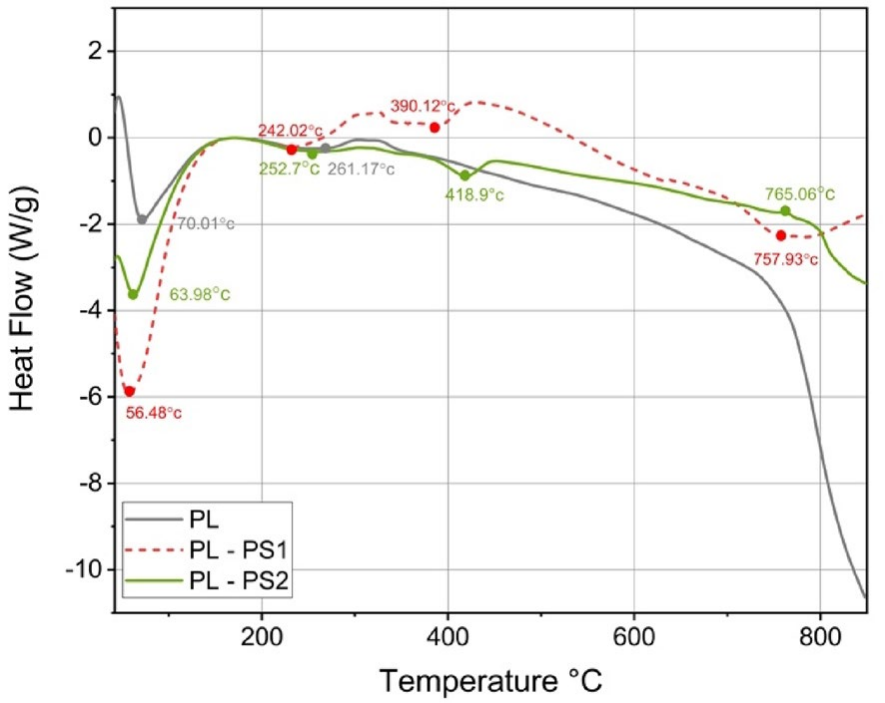
Differential scanning calorimetry (DSC) curves of the BP, BP-PS1 and BP-PS2.

Differential Scanning Calorimetry (DSC) analysis was used to determine the thermal energy absorbed or released via chemical reactions of the constituents of the composite during heating. The DSC thermograms of BP, BP-PS1, and BP-PS2 are shown in Fig. 8, and the results are summarized in Table 6. The DSC curves of BP, BP-PS1, and BP-PS2 show similar patterns with a series of transitions in the curves: firstly, the endothermic glass transition peaks (Tg) at a temperature of 70.01 °C, 56.48 °C. and 63.98 °C for BP, BP-PS1, and BP-PS2 respectively. These peaks corresponded to the evaporation of the fiber moisture content. Secondly, endothermic crystallization transition peaks (Tc) at 261.17 °C. 242.02 °C and 252.7 °C for BP, BP-PS1, and BP-PS2 could be attributed to the degradation of hemicellulose and α-cellulose in the banana peels. Thirdly, endothermic melting transition peaks (Tm) around 750 °C could be attributed to partial lignin degradation^37^. These results agreed with the weight loss in the thermogravimetric analysis of different composites^50^.

**Table 6:** Differential Scanning Calorimetry (DSC) analysis results of the BP, BP-PS1, and BP-PS3.

Samples		T_g_ (°C)	T_c_ peak (°C)		T_m_ peak (°C)
BP	Value	70.01	261.17		–
	ΔH(j/g)	648.2	58.85		–
	Nature of peak	Endothermic	Endothermic		–
BP-PS1	Value	56.48	242.02	390.12	757.93
	ΔH(j/g)	1577	158.0	108	307.1
	Nature of peak	Endothermic	Endothermic	Endothermic	Endothermic
BP-PS3	Value	63.98	252.7	418.9	765.06
	ΔH(j/g)	168.1	55.07	68.02	7.01
	Nature of peak	Endothermic	Endothermic	Endothermic	Endothermic

The crystallization temperatures (Tc) of BP-PS1 and BP-PS2 composites were separated into double peaks (Fig. 8). The reason could be that adding polystyrene to the fibers caused heterogeneous crystal formation^51^. As a result, the crystallization and melting temperatures of the BP-PS1 and BP-PS2 composites were lower than their corresponding values for pure banana peel. A good explanation was suggested by^52^, who related these findings to the formation of tiny crystals due to the strong nucleation on the surfaces of fibers and the consequent shortened time of Ps crystallization. The lower melting peak recorded in the current study was probably caused by the tiny crystals formed by the strong nucleation on the fiber's surfaces and the consequent shortened time of PS crystallization^52^.

## Conclusion

In this study, banana peels and polystyrene were investigated to produce insulating composites. The results demonstrate the potential for developing new recyclable, environmentally friendly materials for building insulation applications. All the bio-composites showed similar thermal conductivity values, ranging from 0.028 to 0.030 W/m.K. In contrast, the pure peel powder offers high thermal stability with 66.4% total weight loss and high crystallinity 56.1%. Based on these findings, replacing conventional thermal insulators with the developed bio-composites will lead to a lower environmental impact by increasing energy efficiency and resource management. However, further studies are required to evaluate the mechanical characteristics and investigate the characteristics required to transform the prepared composites for industrial applications.

## Data Availability

Data will be available upon request. The corresponding author, Irene S. Fahim should be contacted if someone wants to request the data.
